# Estimating cause-specific mortality in Madagascar: an evaluation of death notification data from the capital city

**DOI:** 10.1186/s12963-019-0190-z

**Published:** 2019-07-29

**Authors:** Bruno Masquelier, Gilles Pison, Julio Rakotonirina, Anjarasoa Rasoanomenjanahary

**Affiliations:** 1 Center for Demographic Research, Université catholique de Louvain (UCLouvain) Place Montesquieu, 1, bte L2.08.03, B-1348 Louvain-la-Neuve, Belgium; 2 Institut National d’Etudes Démographiques, 133, boulevard Davout, 75020 Paris, France; 3 Muséum national d’histoire naturelle, Musée de l’Homme, 17 place du Trocadéro, 75116 Paris, France; 4 Département Santé Publique, Faculté de Médecine, BP. 375, Antananarivo, Madagascar; 5 Bureau Municipal d’Hygiène de la Commune Urbaine d’Antananarivo, Madagascar, Rue Raketamanga, Isotry, Madagascar

**Keywords:** Mortality, Causes of death, Antananarivo, Madagascar, Death records, Vital registration, Sub-Saharan Africa, Burden of disease

## Abstract

**Background:**

Trends in cause-specific mortality in most African countries are currently estimated from epidemiological models because the coverage of the civil registration system is low and national statistics on causes of death are unreliable at the national level. We aim to evaluate the performance of the death notification system in Antananarivo, the capital city of Madagascar, to inform cause-of-death statistics.

**Methods:**

Information on the sex of the deceased, dates of birth and death, and underlying cause of death were transcribed from death registers maintained in Antananarivo. Causes of death were coded in ICD-9 and mapped to cause categories from the Global Burden of Disease 2016 Study (GBD). The performance of the notification system was assessed based on the *Vital Statistics Performance Index*, including six dimensions: completeness of death registration, quality of cause of death reporting, quality of age and sex reporting, internal consistency, level of cause-specific detail, and data availability and timeliness. We redistributed garbage codes and compared cause-specific mortality fractions in death records and estimates from the GBD with concordance correlation coefficients.

**Results:**

The death notification system in Antananarivo performed well on most dimensions, although 31% of all deaths registered over the period 1976–2015 were assigned to ICD codes considered as “major garbage codes” in the GBD 2016. The completeness of death notification, estimated with indirect demographic techniques, was higher than 90% in the period 1975–1993, and recent under-five mortality rates were consistent with estimates from Demographic and Health Surveys referring to the capital city. After redistributing garbage codes, cause-specific mortality fractions derived from death notification data were consistent with GBD 2016 for the whole country in the 1990s, with concordance correlation coefficients higher than 90%. There were larger deviations in recent years, with concordance correlation coefficients in 2015 at 0.74 (95% CI 0.66–0.81) for men and 0.81 (95% CI 0.74–0.86) for women.

**Conclusions:**

Death notification in Antananarivo is a low-cost data source allowing real-time mortality monitoring, with a potential to improve disease burden estimates. Further efforts should be directed towards evaluating data quality in urban centers in Madagascar and other African countries to fill important data gaps on causes of death.

**Electronic supplementary material:**

The online version of this article (10.1186/s12963-019-0190-z) contains supplementary material, which is available to authorized users.

## Background

Timely and reliable data on mortality trends and leading causes of death are critical to develop public health policies and monitor health progress. However, civil registration and vital statistics (CRVS) systems are deficient in many low- and middle-income countries. Globally, a little over one third of all deaths are registered worldwide [1]. Madagascar is no exception; its CRVS system is still inoperative at the national level, despite being one of the oldest systems in place in Africa, established in the late nineteenth century [2]. Over the period 1965–1967, about half of male deaths and two-thirds of female deaths were registered [3], but it is unclear whether the system has improved or deteriorated since. The national statistical office ceased to publish estimates based on registration data in the 1970s. Levels and trends in mortality have since been estimated from large-scale sample surveys such as Demographic and Health Surveys (DHS) or from the population censuses (with a long intercensal period between 1993 and 2018). Statistics on causes of death are available from the National Health Information System, but these statistics from health facilities cannot be considered representative of the general population, because they do not include home deaths. As a result, our knowledge of cause-specific mortality in Madagascar is almost entirely based on epidemiological models such as the Global Burden of Disease Study (GBD) [4].

This situation is common to most African countries, where death registration systems are regularly described as rudimentary. In their global assessment of vital statistics, Mikkelsen and colleagues (2015) [1] classified Madagascar among countries with the weakest systems. However, assessments are usually conducted at the national level, and they should be complemented by a detailed examination of the situation prevailing in major urban areas because some cities have functional death registration systems which could inform cause-of-death statistics. Unlike in rural areas, burial permits are often required in urban centers, and this encourages the notification of deaths and their registration. As early as the 1970s, several studies emphasized the promise of death registration in African cities for monitoring mortality trends. In Bamako (Mali), Fargues and Nassour (1988) estimated that the completeness of death registration above age 5 was 73% for men and 68% for women in the late 1970s [5]. In Libreville (Gabon), Antoine et al. (1976) analyzed registration data for the period 1969 to 1972 and obtained reliable mortality rates [6]. In Abidjan (Côte d’Ivoire), a study over the period 1973–1992 showed that death registration was nearly complete for adults, but incomplete for children [7]. In St. Louis (Senegal), death registration was estimated to be at least 90% complete as early as the 1980s [8]. However, these studies are somewhat dated and there have been few recent attempts to measure the performance of death registration systems and the quality of cause-of-death data in African cities.

In this paper, we assess the quality of death notification in Antananarivo, the capital city of Madagascar. Deaths occurring in the six central districts of the city must be notified to the Municipal Office of Hygiene (BMH for Bureau Municipal d’Hygiene). The BMH issues burial permits, but does not officially register deaths; relatives need to report the deaths to the vital statistics office. All records were transcribed from registers maintained by the Municipal Office of Hygiene to build a database covering the period from 1976 to 2015. Previous research analyzed this database to document the mortality crisis that affected the country in the mid-1980s [9] or to provide an overview of trends in all-cause and cause-specific mortality [2, 10]. In this study, we evaluate the quality of the data based on a synthetic index called the *Vital Statistics Performance* (*VSP*) *Index*, developed by Phillips and colleagues [11]. The VSP index has six dimensions: quality of cause of death reporting, quality of age and sex reporting, internal consistency, completeness of death registration, level of cause-specific detail, and data availability and timeliness. We show that the system in place in Antananarivo performs reasonably well on these six dimensions.

The VSP index mainly focuses on the characteristics of the data but does not examine the validity of registered causes of death. Validation studies are very complex, and in Madagascar, there is no solid, independent validation reference to evaluate death notification or registration. In the absence of such reference, we compare cause-specific mortality fractions (CSMFs) with estimates from the GBD 2016 Study. Because GBD estimates refer to the entire country, we expect some differences with death notification from the capital city, but the nature of these deviations can be anticipated. The capital city is better equipped with health facilities than the rest of the country and benefits from a favorable location in the central highlands of Madagascar resulting in few autochthonous malaria cases (malaria is endemic in coastal areas). Access to basic amenities such as sanitation and improved water sources is also more common in Antananarivo. For example, according to the 2008–2009 DHS, 43% of households in Madagascar had no toilet facility, while this proportion was only 1% in the capital city. We can, therefore, expect a more rapid progression of the epidemiological transition in the capital, as compared to the entire country, resulting in higher levels of life expectancy and a more pronounced shift towards non-communicable diseases. CSMFs might also differ simply because the age structure of the population of the capital city could be different from the national age structure, but this can be accounted for through standardization.

This comparison between GBD estimates and death notification data in Antananarivo is not equivalent to validating independent data series. Estimates extracted from the death notification system in place in Antananarivo and covering the period 1984–1995 have been published previously [10], and these were used to model causes of death in Madagascar in the GBD Study [4]. Because these estimates were flagged as non-representative of the country, the variance associated with these data points was increased in the GBD. However, to our knowledge, no specific adjustment was made to account for the fact that they refer only to the capital city. After 1995, GBD estimates for Madagascar are largely model-driven. Several epidemiological studies (related to specific diseases such as hepatitis A, B, and C or meningitis) are used, in combination with police records, results from the National Program for the Fight Against Malaria, and data from DHS (for pregnancy-related mortality). According to an approach called *Cause of Death Ensemble modelling* (Codem), the distribution by cause of death is predicted based on several series of statistical models [12]. A large array of covariates is involved in these models, including the frequency of alcohol consumption, the average body mass index, or the use of contraceptives. Hence, cause-specific mortality fractions extracted from death records in Antananarivo should be consistent with GBD up to the mid-1990s and might potentially deviate more from GBD estimates in recent years.

## Methods

### Data

The *Municipal Office of Hygiene* of Antananarivo was created in 1916 to offer free consultations, conduct immunization campaigns, manage prophylaxis against malaria, and isolate patients with highly infectious diseases. Since 1921, the BMH is also in charge of the verification of all home deaths. Historically, this verification included liver and lung punctures because it was part of prevention measures against the plague, a disease that has marked the history of the city, with several outbreaks, including in 2017. At present, ten doctors are responsible to meet a relative of the deceased and establish the cause of death based on information provided by the family and available medical documents. For deaths that occur in hospitals (about 40% of all deaths since 1976), relatives present to the BMH the death certifying form obtained from the hospital, which includes information on the cause of death. All deaths are recorded in the same register in the BMH. The death report form used by the BMH is reproduced in the Additional file 1. In health facilities, there is a lack of standardization of death certificates and the WHO International Form of Medical Certificate of Cause of Death is not used systematically.

The anonymous data transcribed from registers maintained by the BMH contain the following items: (1) date of birth, (2) gender, (3) date and time of death, (4) date and time of reporting of death, (5) *fokontany* of the deceased (the smallest administrative unit in Madagascar), (6) place of death (name of the hospital, home or street), and (7) diagnosis of the cause of death. Deceased persons whose *fokontany* was outside the capital city were considered as non-residents of the capital and were excluded for this study. The office covers the six central districts of Antananarivo-Renivohitra, corresponding to about 1 million inhabitants in 2009, according to a provisional count made in preparation of the national census (postponed until 2018 due to political instability). The database contains about 315 000 deaths for the period 1976–2015 (excluding stillbirths), with 250 000 deaths of residents. About 82 000 deaths occurred in children under age 15.

Deaths that took place in medical facilities are sometimes reported directly to the BMH with a code corresponding to the 9th revision of the International Statistical Classification of Diseases (ICD), as provided by the medical personnel. Other deaths, for which a cause of death was noted in plain text in the register, or for which a code corresponding to another revision of the ICD was available, were coded using ICD-9 by one physician to construct the database used for this study. ICD-9 codes were then mapped to the different categories of causes included in the GBD 2016 [4].

### Evaluating the performance of the notification system

We slightly adapted the indicators of the *Vital Statistics Performance Index* for our case study [11].

#### Quality of cause of death reporting

One of the key contributions of the GBD Study is the effort to address the problem of garbage coding, that is, the coding of some deaths to uninformative codes. This includes not only causes identified as “undefined” in the specific ICD chapters, but also deaths attributed to causes which should not be considered as initial causes [13]. For example, respiratory failure and pulmonary embolism should not be considered as initial causes of death; they have an underlying cause that precipitated the chain of events leading to death.

To obtain a list of acceptable codes, we used the GBD 2016 cause list as our reference. This list distinguishes 4 different levels. The three level 1 causes are the following categories: (a) *communicable*, *maternal*, *neonatal*, *and nutritional diseases*, (b) *non*-*communicable diseases*, and (c) *injuries*. These categories are then split into 21 level 2 causes: for example, all cancers are assembled together into *neoplasms*. This category is further split into level 3 causes, including cancer sites such as *lip and oral cavity cancer*. We used the level 3 causes as our most refined disaggregation, disregarding level 4 (e.g., the distinction between liver cancers due to hepatitis B, hepatitis C, alcohol use, or other causes).

Detailed mapping of ICD-9 codes to GBD 2016 cause categories is provided by Naghavi and colleagues (2017) [4]. This list also identifies garbage codes in four levels, corresponding to the GBD cause hierarchy: GBD level 1 garbage codes refer to ICD codes which should be redistributed across likely causes of deaths that span the three broad levels 1 of the GBD cause list. For example, there is not enough information for deaths coded *heart failure unspecified* (428.9 in ICD-9), and these could be redistributed across communicable, non-communicable diseases, and injuries. GBD level 2 garbage codes refer to cases for which an acceptable level 1 cause can be assigned. For instance, deaths coded 578.9, corresponding to *hemorrhage of gastrointestinal tract*, *unspecified*, can be redistributed only within non-communicable diseases. For GBD level 3 garbage codes, the redistribution can take place only within one of the 21 broad categories that correspond to GBD level 2 causes. As an example, deaths coded *malignant neoplasm of uterus*, *part unspecified* (179) can be reassigned a likely cause of death within neoplasms. The final list of garbage codes, such as *acute but ill-defined cerebrovascular disease* (436), refers to cases where the redistribution can occur within a level 3 cause (in this example, *cerebrovascular disease*).

To assess the quality of cause of death reporting in the registers of Antananarivo, we computed the percentage of deaths assigned to ICD-9 codes identified as major garbage codes, that is, GBD levels 1 and 2 garbage codes.

#### Quality of age and sex reporting

When evaluating the quality of age and sex data, Phillips and colleagues (2014) use as an indicator the proportion of deaths for which the sex or age of death is unspecified. Here, we computed the share of deaths notified in Antananarivo for which the age is known to the day and to the month.

#### Internal consistency

The third dimension refers to the proportion of deaths where the underlying cause is consistent with the age at death and/or sex of the deceased. For example, the death of a child under age 15 cannot be associated with some types of neoplasms (e.g., lung or larynx), and a woman cannot die from prostate cancer. To calculate the proportion of inconsistent causes of death, we used a list established by Phillips et al. (2014). Causes of death flagged as incompatible with ages at death or sex were later reclassified as garbage codes and redistributed.

#### Completeness of death registration

Demographers regularly measure the completeness of death registration through indirect techniques known as death distribution methods (DDMs). These methods compare the age distribution of deaths between two censuses with the age distribution of the population enumerated in these censuses in order to estimate the fraction of deaths that are reported. We applied the generalized growth balance method (GGB) [14] and the synthetic extinct generation method (SEG) [15] based on the age distribution of the population registered in 1975 and 1993 and the age distribution of notified deaths between the two censuses. These methods are based on strict assumptions: (1) that the population is closed to migration, (2) that the completeness of reporting of the population and deaths are invariant by age (beyond a certain age), and (3) that there are not systematic age errors. In the absence of data to make adjustments, we assumed that net migration in the central districts of Antananarivo is negligible. Compared to other African capitals, Antananarivo is characterized by a low rate of growth (estimated at 4.7% per year over the period 1975 to 1995) and relatively constant immigration rates [16, 17]. The few available means to make sure that the assumptions underlying the GGB and SEG methods are verified are visual checks based on diagnostic plots [18, 19]. At the time of writing, the results from the 2018 census were not available. To assess completeness in more recent years, we used another method, developed by Adair and Lopez (2018), based on regression models to predict completeness of death registration from the crude death rate, the level of under-five mortality rate, and the population age structure, informed by estimates from the GBD Study [20]. To estimate completeness of death reporting in children, we also computed under-five mortality rates from full birth histories collected in Demographic and Health Surveys, extracting from the datasets only the records of women interviewed in the capital city, and pooling all surveys together. Mortality rates were obtained based on a synthetic cohort approach [21]. We compared trends in child mortality from death notification and DHS estimates. Finally, we compared mortality rates derived from death records with that of the GBD 2016 Study for the whole country. We estimated the population at risk of dying based on a mixture of administrative counts, censuses and sample surveys (see Additional file 1).

#### Level of detail on causes of death

The list of causes of death available through the registration system should be sufficiently disaggregated to be useful for decision-making in the health sector. Phillips et al. (2014) calculate the percentage of causes considered in the GBD 2010 study (192 single causes) that are mentioned in the death registers. However, there is no reason to include causes that never occurred in the country, such as Ebola or Zika. The GBD 2016 Study estimated that deaths in Madagascar from 1990 to 2016 have been caused by 139 different level 3 causes of death. Since it is not possible to map ICD-9 codes to GBD causes for 7 of these distinct causes,^1^ the list is reduced to 132 causes for this study. The level of detail on causes of death is computed as the number of distinct causes reported in Antananarivo, divided by 132.

#### Data availability and timeliness

For the last dimension of the Vital Statistics Performance Index, Phillips et al. (2014) use a weighted smoothing algorithm that gives more weight to recent data over data produced intermittently or not updated recently. Here, we simply note that data are continuously generated and readily available for analysis upon request. Deaths should normally be reported within 12 days. Over 80% of deaths reported to the BMH took place on the same day or the day before.

### Comparison with the GBD estimates of cause-specific mortality

Comparing cause-specific mortality fractions in GBD and in death records requires correcting for garbage coding in the registers. As part of the GBD, different algorithms are used for garbage code redistribution (based on fixed proportions, regression models, and proportional reassignment and based on multiple causes of death data). Most of the code used in the GBD 2016 Study for the garbage code redistribution is distributed in the public domain. However, to our knowledge, the full list of target codes (i.e., valid causes) for each garbage code is not yet available. In addition, garbage code redistribution is an integral part of the estimation process of the GBD Study, and it is difficult to replicate only this segment of the analysis as an independent exercise on a specific database. Therefore, in order to map all ICD codes to acceptable GBD cause categories, we used here a simplified garbage code redistribution algorithm.

We proceeded in seven steps. First, all ICD-9 codes initially mapped to level 4 garbage codes were assigned to an acceptable level 3 cause. For example, all deaths coded *bronchopneumonia*, *organism unspecified* (485) were assigned to *lower respiratory infections*. Second, following the World Health Organization (WHO) and earlier versions of the GBD Study [22], we redistributed ill-defined cardiovascular diseases to *ischemic heart disease* and *other cardiovascular and circulatory diseases* using age-specific correction factors [23]. Third, ill-defined neoplasms were redistributed pro rata across all cancer sites identified in GBD within each age group and sex (except liver, pancreas, ovary, and trachea, bronchus, and lung cancer). Fourth, ICD-9 codes from Chapter XVI (symptoms, signs and ill-defined conditions) were redistributed pro rata across all GBD causes (excluding injuries) within each age group, sex, decade, and season of death. There are strong weather variations in Antananarivo, and mortality rates from infectious diseases and nutritional deficiencies in children peak in the hot and rainy season (from November to April), while mortality rates in adults from cardiovascular diseases and diseases of the respiratory system peak in the dry season. Fifth, we redistributed pro rata by age and sex injuries undetermined whether accidentally or purposefully inflicted and all ICD-9 codes from Chapter XVII (injury and poisoning) to the GBD categories for intentional and unintentional injury. Sixth, for all remaining level 3 garbage codes represented in our database, we identified the most likely level 2 cause (e.g., *neoplasms*). The ICD-9 tagged as garbage codes were redistributed pro rata by age, sex, and year to level 3 causes within each level 2 cause. Finally, all garbage codes that had not been redistributed in earlier steps were redistributed pro rata by age, sex, season, and decade of death across all causes of death. Further details on this approach to garbage code redistribution are provided in  Additional file 1.

After redistributing garbage codes, we compared ranks of leading causes of death in 1990 and 2015. To control for variations in the age structure, death records from Antananarivo were weighted to impose the age pattern of deaths estimated in the GBD in Madagascar. Finally, cause-specific mortality fractions from the two sources were assessed for concordance using Lin’s concordance correlation coefficient [24], measured annually for each sex. GBD estimates were obtained from the Global Health Data Exchange (GHDx) website [25].

## Results

### Quality of cause of death reporting

Over the period 1976–2015, 23.8% of all deaths in registers from Antananarivo were attributed to a level 1 garbage code and 7.6% to a level 2 garbage code. The top 10 causes most frequently attributed to a major garbage code are listed in Table 1. Together, these 10 causes represent 18.6% of deaths in the period 1976 to 2015.

**Table 1:** Top 10 ICD-9 codes flagged as “major garbage codes” in the mortality database of Antananarivo (1976–2015), according to the GBD classification

Rank	ICD-9 codes	% of deaths	Code description	Garbage code level	Listed as *garbage code* by the WHO
1	428.9	5.9	Heart failure, unspecified	1	Yes
2	797	3.0	Senility	1	Yes
3	799.9	2.9	Other unknown and unspecified cause of mortality	1	Yes
4	785.5	1.8	Shock without mention of trauma (symptoms involving cardiovascular system)	1	Yes
5	428	1.0	Congestive heart failure, unspecified	1	Yes
6	586	1.0	Renal failure, unspecified	1	No
7	799.1	0.9	Respiratory arrest	2	Yes
8	578.9	0.8	Hemorrhage of gastrointestinal tract	2	No
9	276.5	0.7	Dehydration	1	No
10	38.9	0.7	Septicemia	1	No
	Total	18.6%			

**Fig. 1:**
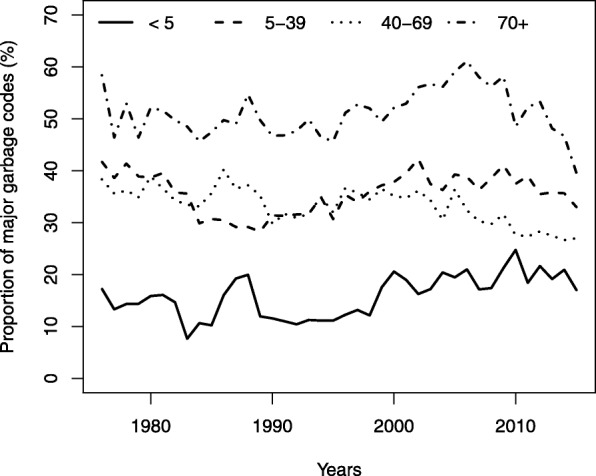
Proportions of major garbage codes (levels 1 and 2) by age group in the mortality database of Antananarivo (1976–2015)

The proportion of deaths assigned to major garbage codes varies considerably by age, from 14% in children aged 0–4 to 51% among adults who died at age 70 or above (Fig. 1). By contrast, it varies relatively little over time. The small peak in the second half of the 1980s in children and adults aged 40 and above can be attributed to the acute health crisis due to a resurgence of malaria and malnutrition [9], which could have disrupted the organization of health services and the *Municipal Office of Hygiene*.

### Quality of information about age and sex

Sex is reported for over 99% of all deaths. The year of birth and death is also known for virtually all reported deaths since 1976. However, the accuracy of dates of birth and death varies over time. In 1976, the age at death was known up to the day in 59% of cases. This proportion dropped to 41% in 1981, again most likely due to the health crisis, then increased gradually to 85% in 2001. In the most recent years, it is possible to calculate the age at death up to the day for over 99% of deaths.

### Internal consistency of data

In 98.9% of deaths (excluding those assigned to garbage codes), the GBD category of causes of death is consistent with the age and sex of the deceased. In 0.6% of cases, the age at death is too young for the assigned cause. This is mostly the case of deaths among infants and classified as due to chronic obstructive pulmonary disease, originally coded in ICD-9 as bronchitis (490, 491.8). In 0.3% of deaths, the inconsistency is due to *neonatal sepsis and other neonatal infections* assigned to deaths that occurred in the post-neonatal period.

### Completeness of death registration

**Fig. 2:**
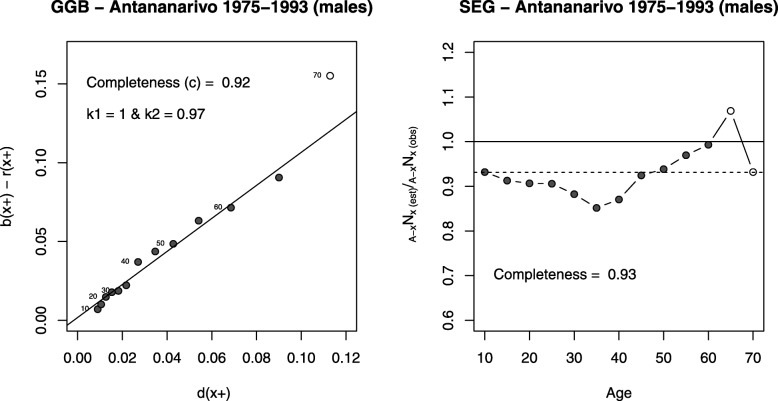
Diagnostics plots of the GGB and SEG method for male deaths between 1975 and 1993

Figure 2 shows, for men, the two diagnostic plots for the GGB and SEG methods. In the case of the GGB, the estimated completeness is 92% (the same value is obtained for women). The population appears underestimated by 3% in the 1993 census, as compared to the 1975 census (7% in women). With the SEG method, we obtain a similar estimate of completeness, 93% for both sexes. The completeness estimate obtained from the Adair & Lopez method (model 2) is 93% for males and 92% for females for the whole period (1976–2015), which is remarkably consistent with results from the death distribution methods for the period 1975–1993. Based on these analyses, we adjust deaths based on an estimate of the completeness of 92.5% for both sexes to estimate mortality rates (the average between estimates of the SEG and GGB methods).

**Fig. 3:**
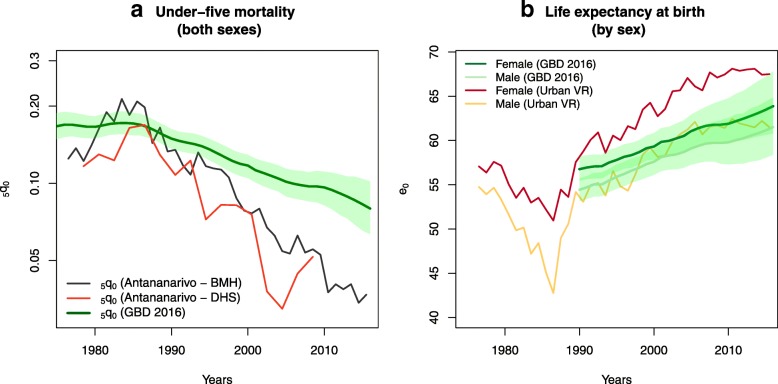
Trends in child mortality and life expectancy at birth in Antananarivo according to death notification data and GBD 2016.

Trends in child mortality (for both sexes) from death notification data are compared with GBD 2016 estimates and DHS mortality rates for the capital city in Fig. 3a [25]. Child mortality rates estimated for Antananarivo are lower than GBD estimates, but this pattern is expected, due to the city’s more advantageous health and economic development. Estimates from death notification depict a similar rate of reduction as in DHS, but at a slightly higher level, indicating good completeness of registration of deaths in children. Figure 3b displays trends in life expectancies from GBD and estimates from death records. The health crisis in the 1980s is instantly recognizable in these trends. A resurgence of infectious diseases, respiratory, and nutritional deficiencies resulted in a drop in life expectancy of 12 years for men (7 years for women) in the capital [2, 9]. Since 1990, both sources indicate that life expectancy has increased gradually, but levels differ. For men, estimates derived from death notification are close to GBD life expectancies and contained within the GBD 95% uncertainty intervals during most of the 1990–2015 period. By contrast, life expectancy levels among females are always higher than GBD estimates and above the 95% uncertainty intervals.

### Level of cause-specific detail

Death notification data provide information on 128 of the 132 causes of death (level 3) apparent in GBD estimates for Madagascar. The four causes that are not mentioned in the records are *dengue fever*, *iodine deficiency*, *cystic echinococcosis*, and *conflict and terrorism*. The level of detail has improved over time, from about 95 different causes reported annually before 1980 to about 102 annually since 2010.

### Leading causes of death

**Fig. 4:**
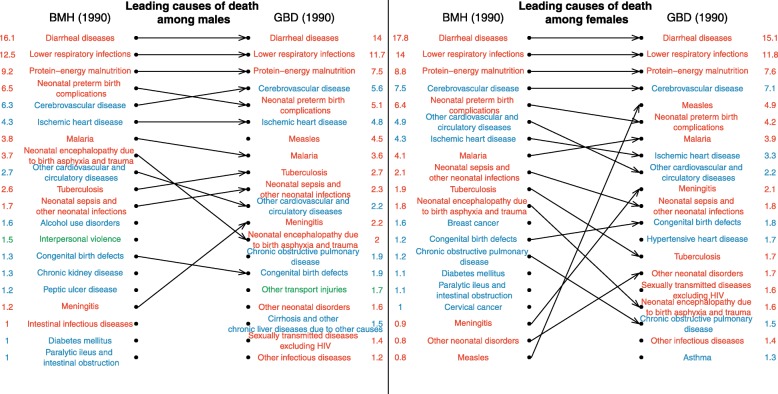
Comparison of the ranking of causes of deaths according to death registers from Antananarivo and the GBD 2016, for 1990. Cause-specific mortality fractions in columns are in %. Estimates from death records are standardized using the age structure of deaths estimated in the GBD 2016 in 1990.

The ranking of the top 20 causes of death in 1990 is presented in Fig. 4 for each sex. Cause-specific mortality fractions (CSMFs) from both sources are shown in columns. To be compared with GBD estimates, the CSMFs from death records were standardized using the age structure of deaths estimated in the GBD 2016 in 1990 and 2015. The unstandardized cause-specific mortality fractions for the capital city are displayed in Additional file 1. Among men, the six leading causes of death in 1990 according to GBD 2016 correspond to the top six causes of death according to the death records. The four leading causes of deaths among women are also the same in both sources. The overall concordance correlation coefficient between CSMFs from death records and GBD 2016 is 0.96 (95% CI 0.94 to 0.97) for men in 1990 and 0.95 for women (95% CI 0.94 to 0.96). The largest difference in terms of percentage of deaths assigned to a specific cause relates to measles, which represents 4.5% of male deaths (95% UI 1.7–9.4) and 4.9% of female deaths (95% UI 1.9–10.1) according to the GBD, but caused only 0.5% of male deaths and 0.8% of female deaths in the registers in 1990. However, there is substantial uncertainty around annual estimates for measles due to its epidemic nature. In terms of broad categories of causes of death, there is a very high consistency between estimates in 1990 (Table 2), reflecting the fact that GBD estimates were modeled based on the same data from the capital in this period.

**Table 2:** Percentage of deaths due to communicable, maternal, neonatal, and nutritional diseases, non-communicable diseases, and injuries according to GBD 2016 estimates and death records in 1990 and 2015

	1990				2015			
	Males		Females		Males		Females	
	GBD	Death records	GBD	Death records	GBD	Death records	GBD	Death records
Communicable, maternal, neonatal, and nutritional	63 (61–66)	63	66 (63–69)	64	54 (48–59)	38	53 (48–60)	36
Non-communicable	31 (28–33)	31	30 (27–33)	33	39 (34–44)	53	42 (36–47)	59
Injuries (including self-harm and interpersonal violence)	6 (5–7)	6	4 (3–4)	3	7 (6–8)	8	4 (4–5)	4

Cause-specific mortality fractions are in % (with 95% uncertainty intervals for the GBD in parentheses). Estimates from death records are standardized using the age structure of deaths estimated in the GBD 2016 in 1990 and 2015

**Fig. 5:**
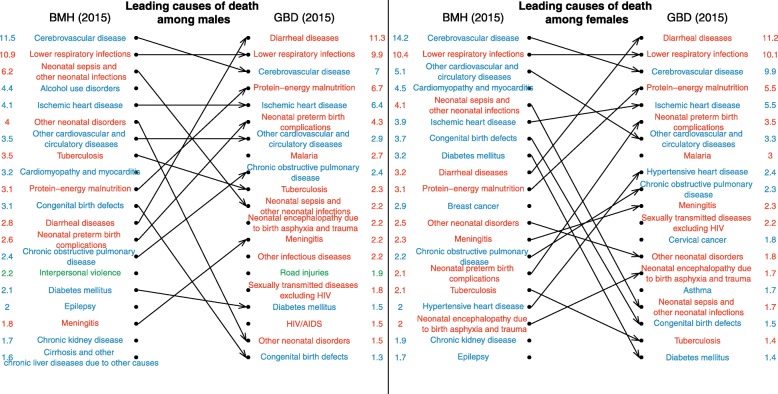
Comparison of the ranking of causes of deaths according to death registers from Antananarivo and the GBD 2016, for 2015. Cause-specific mortality fractions in columns are in %. Estimates from death records are standardized using the age structure of deaths estimated in the GBD 2016 in 2015.

Deviations between estimates from GBD and those derived from death registers increase over time, especially once death notification data are no longer available to inform the models (after 1995). The concordance correlation coefficients remain higher than 90% until 1999, but they decline to 0.87 for men (95% CI 0.82–0.91) and 0.84 for women (95% CI 0.79–0.89) in 2000. In 2015, they amount to 0.74 (95% CI 0.66–0.81) for men and 0.81 (95% CI 0.74–0.86) for women. Figure 5 shows the rankings of the leading causes of death in 2015. In both sources, and for both sexes, lower respiratory infections are still the second most important cause of death. However, as a result of the more advanced epidemiological transition, cerebrovascular diseases have become the first cause of death in the capital city, according to death records. By contrast, diarrheal diseases still rank first in GBD estimates, causing more than 11% of deaths (95% UI 7.7–14.8) in both sexes, while according to death registers, they caused about 3% of deaths in the capital city, and now rank 12th in men and 9th in women. Other leading causes of death with higher rankings in the GBD for both sexes include protein-energy malnutrition, neonatal preterm birth complications, and malaria. Overall, in 2015, the percentage of deaths due to communicable, maternal, neonatal, and nutritional diseases is 54% (95% UI 48–59) among men in GBD estimates, against 38% in death records (Table 2). The percentage among women is 53% in GBD (95% UI 48–60), against 36% in death records. Conversely, the percentage of deaths due to non-communicable diseases is lower in GBD estimates (e.g., 39% (95% UI 34–44) in men against 53% in death records). There is a good agreement between sources in the share of violent deaths (e.g., 7% (95% UI 6–8) in men against 8% in death records).

## Discussion

Outside of South Africa, death registration systems in sub-Saharan Africa are deemed rudimentary or non-existent, because most assessments are conducted at the national level. Due to the lack of registration data, cause-specific mortality rates are mainly estimated based on epidemiological models by UN agencies and academic groups, such as the Institute for Health Metrics and Evaluation (IHME), which leads the Global Burden of Disease Study. For data-sparse countries, the World Health Organization now largely resorts to GBD estimates to obtain cause-specific mortality fractions [22]. The GBD Study uses all the data available, including verbal autopsies studies in countries with incomplete vital registration, sometimes at the subnational level. However, persistent data gaps remain, and innovative ways to reduce these gaps are needed.

In this study, we evaluated the performance of the death notification system in place in Antananarivo. We estimated that the completeness of death reporting among adults was higher than 90% in the period 1975–1993. Recent estimates of under-five mortality are consistent with trends derived from Demographic and Health Surveys and even provide higher mortality rates than in DHS. We also showed that life expectancies computed from death notification data are similar to estimates in the GBD 2016 study for men (they fall within the 95% confidence intervals in the GBD), but we observed larger deviations in the levels of female life expectancy. In the period 1990 to 2015, the average difference between the female life expectancy according to GBD and that obtained from death notification was 4.4 years, against only 0.9 years among men. We can reasonably assume that life expectancies are higher in Antananarivo than in the whole country, so these differences go in the expected direction. However, differences by sex in the agreement between series could point to some level of underreporting of female deaths in the death notification system, or to low male-to-female sex ratios of mortality in the GBD, or a combination of both.

The system also performs well on four other dimensions of the VSP index: quality of age and sex reporting, internal consistency, level of cause-specific detail, and data availability and timeliness. However, in terms of quality of cause of death reporting, an important proportion of deaths were assigned to major garbage codes (31% over the period 1976–2015). This proportion rises to 51% among older adults aged 70 and above. It is worth noting that the list of garbage codes in the GBD is very large, and some of these codes, such as 586 (*unspecified renal failure*), are not identified as garbage codes by the WHO [22].

Phillips and colleagues (2014) developed a procedure to combine the different dimensions detailed above in a composite Vital Statistics Performance Index (VSPI) [11]. Applied at the national level, this approach puts Madagascar among the countries with the weakest systems (VSPI below 0.25) [1]. Yet, our own assessment of death notification data from Antananarivo provides a much higher value of the composite index, estimated at 0.52 (Additional file 1). This corresponds to levels attained at the national scale by countries such as Switzerland, Turkey, Iran, or Peru in 2005–2012 [1]. Apart from South Africa and Egypt, no country in Africa had reached such high scores. Lowering the percentages of deaths assigned to garbage codes (especially ill-defined cardiovascular diseases, ICD-9 codes for symptoms, signs and ill-defined conditions, or injuries undetermined whether accidentally or purposefully) is the main way to improve the system in place in Antananarivo.

Despite this high percentage of garbage codes, after redistribution, we observed strong concordance between cause-specific mortality fractions in GBD estimates and death records in 1990 (concordance correlation coefficients were higher than 90% in both sexes). This suggests that a simplified algorithm to redistribute garbage codes can provide plausible estimates. Authors of the GBD Study had access to some death counts from the same registers, from 1984 to 1995. Hence, this high concordance is largely a result of GBD estimates being modeled on the basis of notification data during this period, although one would have expected larger differences since data from the capital city should not be considered representative of the national level. Differences between the two series are more pronounced in 2015, with concordance correlation coefficients in cause-specific mortality fractions declining to 0.74 (95% CI 0.66–0.81) for men and 0.81 (95% CI 0.74–0.86) for women. This decline is presumably due to the absence of mortality measurements in the input database for Madagascar in recent years. The epidemiological transition seems to have progressed much faster in Antananarivo than according to GBD estimates for the country. This could reflect real transitions. However, the decline in concordance over time, as the availability of mortality measurements is reduced, also suggests that there remains room for improvement when predicting the distribution of causes of death based largely on covariates.

Our evaluation of death notification data has three main limitations. First, the population at risk of dying is difficult to estimate in the absence of recent census data. Under-five mortality rates and trends in life expectancy inferred from the death notification could be biased if our population estimates are inaccurate. However, this should not affect our observations about gender differences in life expectancy. Madagascar has conducted a new census in 2018, and this new census will help in estimating population exposure for future analysis. Second, deaths associated with garbage codes were redistributed by adopting an approach that differs from redistribution algorithms used in the GBD. More research efforts should be devoted to developing a publicly available list of target codes (i.e., valid causes) for each garbage code and facilitating the redistribution of garbage codes in specific databases without replicating the whole GBD exercise. Third, there is a lack of independent comparison data to assess the validity of the notified causes of death, since GBD estimates are in part informed by the registers from Antananarivo.

## Conclusion

This study illustrates the potential of urban vital registration for real-time mortality monitoring, and improvement of disease burden estimates, provided that biases related to the urban location of the data source are adjusted for.

Although we report on the situation in Antananarivo only, other major cities in Madagascar have a similar system in place, and some have carefully preserved death records (such as in Antsirabe and Fianarantsoa). These records remain largely untapped, and to our knowledge, the quality of data collected in these cities has not been evaluated. There are significant differences in the structures and processes of the municipal health offices across the country, but all offices tend to be in short supply of resources to store the registers and encode them in databases. They also lack the human and financial resources needed to ensure that all home deaths are verified. Initiatives to expand the civil registration system in Madagascar should support these local structures. Feasibility surveys are also needed to assess the potential of verbal autopsies (VA) for deaths that are reported to the offices in other cities but cannot be certified by a doctor. In 2017, Madagascar undertook a comprehensive assessment of its CRVS system, supported by the *Africa Programme for Accelerated Improvement of Civil Registration and Vital Statistics* (APAI-CRVS)^2^. This assessment identified a series of priorities for strengthening the death registration system, such as introducing verbal autopsies with the WHO VA instrument, harmonizing the content of the death certificate used in health facilities and in municipal hygiene offices, and reinforcing the interoperability between these offices and the Ministry of Health. With the support of the WHO and USAID, the Ministry of Health is also piloting DHIS2, an open-source health management information system platform which will help in centralizing and standardizing the management of cause-of-death data, ideally through the adoption of a shortlist of ICD codes (such as the WHO Start-Up Mortality List).

This study is based on registers from one city only, and the system in Madagascar has some specific characteristics that may not be found in other countries. For example, the country has a very long tradition of civil registration dating back to the nineteenth century, encouraging the prompt reporting of deaths. Locally, village and neighborhood chiefs (*fokontany*) are involved in the notification of deaths to the health sector. The repatriation of the deceased to the ancestral tomb plays a key role in the culture of Madagascar, and death certificates will be required to move the bodies of the deceased. In addition, for deaths occurring in cities, burial permits and death certificates are issued only after a physician has established a cause of death, and fines are imposed in case of unauthorized burial. All these factors contribute to high completeness of registration and make this system a relative exception in Africa. However, previous research conducted in other African cities (including Abidjan, Bamako, and Libreville) has shown that functional death registration systems are in place elsewhere. Most initiatives devoted to supporting CRVS systems have focused on assessments at the national level, digitalization of records, advocacy, and training [26]. More efforts should be devoted to conducting evaluation studies at the local level to fill data gaps. Local systems of death registration could potentially also inform cause-of-death statistics and offer good starting points to scale-up CRVS systems.

## Additional file

Additional file 1: Supplemental information on estimation of the population at risk of dying, redistribution of garbage codes, unstandardized cause-specific mortality fractions from death notification data, computation of the composite index, and death report forms used by the Municipal Hygiene Office. (DOCX 794 kb)

## Data Availability

The dataset containing ICD-9 codes and GBD cause categories needed to replicate this analysis are available upon request from the first author.

## Abbreviations

BMH: Municipal Office of Hygiene (of Antananarivo)
CRVS: Civil registration and vital statistics
CSMF: Cause-specific mortality fraction
DDM: Death distribution methods
GBD: Global Burden of Disease Study
GGB: Generalized growth balance method
GHDx: Global Health Data Exchange
ICD: International Statistical Classification of Diseases and Related Health Problems
IHME: Institute for Health Metrics and Evaluation
SEG: Synthetic extinct generation method
VSPI: Vital Statistics Performance Index
WHO: World Health Organization

## Footnotes

1 The GBD cause list distinguishes between cirrhosis and other chronic liver diseases due to hepatitis B and hepatitis C, due to alcohol use, and due to other causes. However, the mapping of ICD-9 codes to GBD causes is only available for the level 2 cause “Cirrhosis and other chronic liver diseases”. We mapped all ICD-9 codes related to cirrhosis (456.0–456.2, 571–571.9, 572.2–573.9) to “Cirrhosis and other chronic liver diseases due to other causes”. Other causes for which no ICD-9 codes are provided are Mesothelioma, Late maternal deaths, Maternal deaths aggravated by HIV/AIDS, and Road injuries (considered here with “other transport injuries”).

2 Comité National de Coordination en charge de l’amélioration des systèmes nationaux d’état-civil et d’établissement des statistiques de l’état-civil (CNC-CRVS), *Rapport d’évaluation des systèmes d’enregistrement des faits d’état-civil et d’établissement des statistiques de l’état civil*, November 2017, available at https://www.unicef.org/madagascar/media/1871/file/Evaluation%20CRVS%202017.pdf, accessed May, 27, 2019
