# Supplementary material for: Estimating cause-specific mortality in Madagascar: an evaluation of death notification data from the capital city
Source: Popul Health Metr. 2019 Jul 29;17:8. doi: 10.1186/s12963-019-0190-z (PMC6664726; doi:10.1186/s12963-019-0190-z)
Supplement: Supplementary file 1 — Supplemental information on estimation of the population at risk of dying, redistribution of garbage codes, unstandardized cause-specific mortality fractions from death notification data, computation of the composite index, and death report forms used by the Municipal Hygiene Office. (DOCX 794 kb) [file 12963_2019_190_MOESM1_ESM.docx]

Additional file

# Estimation of the population at risk of dying

In the absence of a recent census, estimating the population at risk of dying is difficult.  To obtain population counts for the six central districts of Antananarivo covered by the *Municipal Office of Hygiene*, we fit a cubic smoothing spline to administrative counts available from 1901 to 1972, the 1975 and 1993 censuses, and a provisional count conducted in 2009 as part of the preparation of the next census. We obtained a very regular population increase, from 2.5% annually in the period 1975-1993, to 2.4% annually in the period 1993-2009 (Figure S1). A new census was conducted in 2018, but the disaggregated data was not available at the time of the analysis. The age structure of the population was derived by interpolating between the 1975 and 1993 censuses and four DHS surveys (1992, 1997, 2003-04 and 2008-09). Although DHS surveys do no provide population counts, they give a sense of the age and sex composition of the population. All standard DHS conducted in Madagascar used a two-stage stratified sampling design. At the first stage, clusters were systematically selected from a list of enumeration areas obtained from a recent sampling frame. The sampling frame used for the 1992 DHS was obtained as part of the activities carried out in 1991 in preparation for the 1993 census. The 1997 and 2003/2004 surveys used the master sample constructed by the National Statistical Office (INSTAT) on the basis of the 1993 census. Maps and enumerations prepared for the 2008 census (postponed until 2018) provided another sampling frame for the 2008/2009 DHS survey. All samples were stratified by urban and rural status, and in the most recent surveys, they were also stratified by provinces or smaller administrative units. All households in these enumeration areas were listed and maps in the selected clusters were updated. At the second stage, households to be included in the surveys were selected, with the number of households selected per clusters being inversely proportional to the size of the clusters. Within each selected household, information was collected on the age and sex of household members. We identified clusters located in the capital city and extracted the population pyramids of members of households in these clusters. To break down by age and sex our estimates of the population at risk of dying, we used cubic spline functions to interpolate between the proportions of individuals by age and sex from censuses and surveys (Figure S2).


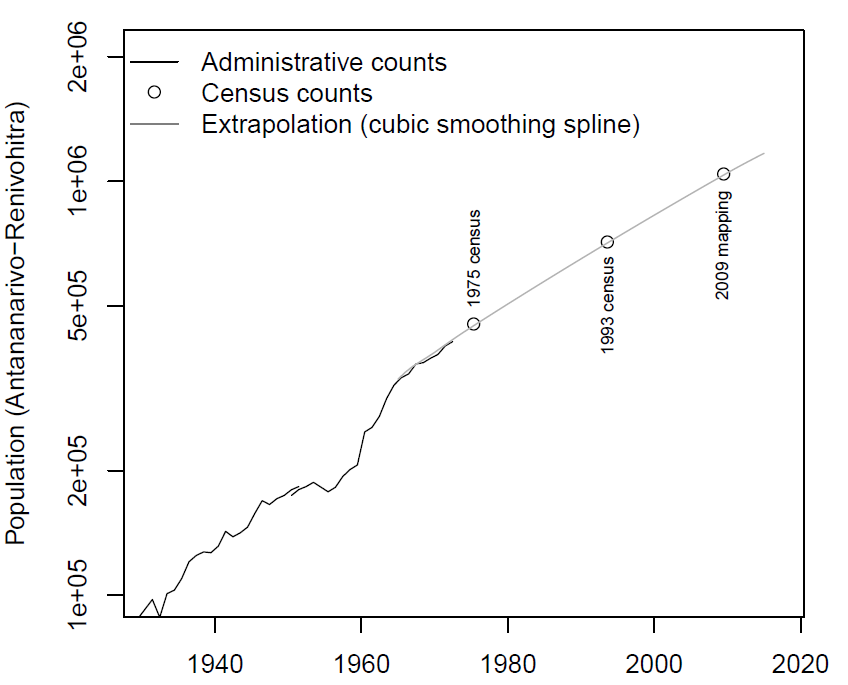


*Figure S1: Population counts in administrative records, census counts, and extrapolated population based on a cubic smoothing spline*


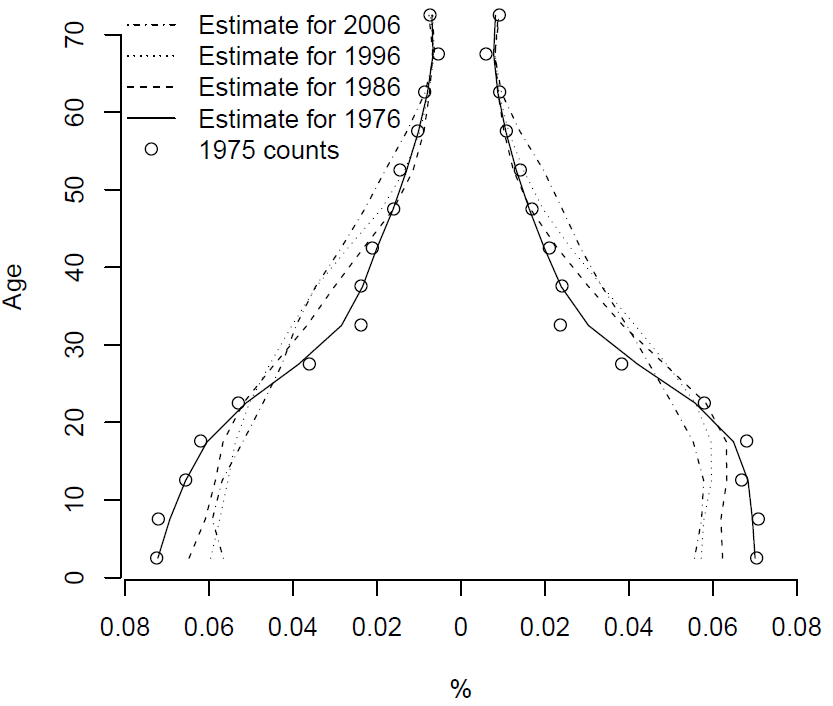


*Figure S2: Changes in the age structure of the population (1975 census counts and estimates from 1976 to 2006)*

# Redistribution of garbage codes

The table below shows how the percentage of deaths assigned to garbage codes declines as deaths are redistributed to acceptable causes of the GBD cause list.

|  | Percentage assigned to | | | |
| --- | --- | --- | --- | --- |
|  | Level 1 garbage codes | Level 2 garbage codes | Level 3 garbage codes | Level 4 garbage codes |
| Before any redistribution | 23.8% | 7.6% | 3.3% | 15.9% |
| STEP 1: ICD-9 codes mapped to Level 4 garbage codes are assigned to a Level 3 cause. The corresponding ICD-9 codes are the following: 084, 084.6, 238, 244, 244.9, 289.8-289.9, 320, 320.9, 425, 425.4,425.9, 429.1, 436-437, 437.9-439.6, 482.9-483, 484, 484.8-486.9,770.0, E808-E829 | 23.8% | 7.6% | 3.3% | 0% |
| STEP 2: Ill-defined cardiovascular diseases are redistributed to *Ischemic heart disease* and *Other cardiovascular and circulatory diseases.* The corresponding ICD-9 codes are 427.1, 427.4, 427.5, 428, 429.0, 429.1, 429.2, 429.9, and 440.9. We used the age-specific correction factors for “High ill-defined coding countries” available in A Lopez et al. (2006) *Global Burden of Disease and Risk Factors*. Oxford University Press and The World Bank, 2006. | 15.6% | 7.6% | 2.4% | 0% |
| STEP 3: Ill-defined neoplasms (ICD-9 codes 195–199) are redistributed pro rata across all cancer sites identified in GBD (except liver, pancreas, ovary, and trachea, bronchus, and lung) within each age group and sex. | 15.6% | 7.6% | 2.1% | 0% |
| STEP 4: ICD-9 from Chapter XVI (symptoms, signs and ill-defined conditions) are redistributed pro rata across all GBD causes (excluding injuries) within each age group, sex, decade of death, and season of death. | 5.2% | 6.5% | 2.1% | 0% |
| STEP 5: Injuries undetermined whether accidentally or purposefully inflicted (ICD-9 codes E980-989) or ICD-9 from Chapter XVII (injury and poisoning) are redistributed pro rata by age and sex to the GBD categories for intentional and unintentional injury. | 5.0% | 2.6% | 2.1% | 0% |
| STEP 6: All remaining ICD-9 codes mapped to Level 3 garbage codes in the GBD 2016 are linked to a Level 2 cause category. They are redistributed pro rata by age, sex, season and year of death to acceptable Level 3 GBD causes within this Level 2 cause category. | 5.0% | 2.6% | 0% | 0% |
| STEP 7: All remaining ICD-9 codes mapped to Level 1 or 2 garbage codes in the GBD 2016 are redistributed pro rata by age, sex, season and year of death to acceptable Level 3 GBD causes. | 0% | 0% | 0% | 0% |

# Unstandardized cause-specific mortality fractions from death notification data

In the main text, we report on cause-specific mortality fractions after standardization, because the age pattern of deaths in the capital city differs from that in the GBD estimates. Differences in the age patterns of deaths are expected, in part because of differences in the age composition of the population at risk of dying. The capital city has an older population than the country as a whole. For example, in the 1993 census, the population aged 0-14 represented 35% of the capital city, against 45% in the country as a whole, due to lower fertility rates in Antananarivo. Trends in mortality will also affect the age pattern of deaths. In particular, under-five mortality has declined much faster in the capital city than in the national average (as captured in GBD). As a result, the fraction of deaths occurring in adult ages will be higher in the death notification data, and there should be fewer under-five deaths. This can be observed below, for two years (1990 and 2015) and each sex. While the age pattern is relatively similar in 1990 (although the percentage of deaths occurring in children under the age of 5 is lower in death notification data), there are substantial differences in 2015.


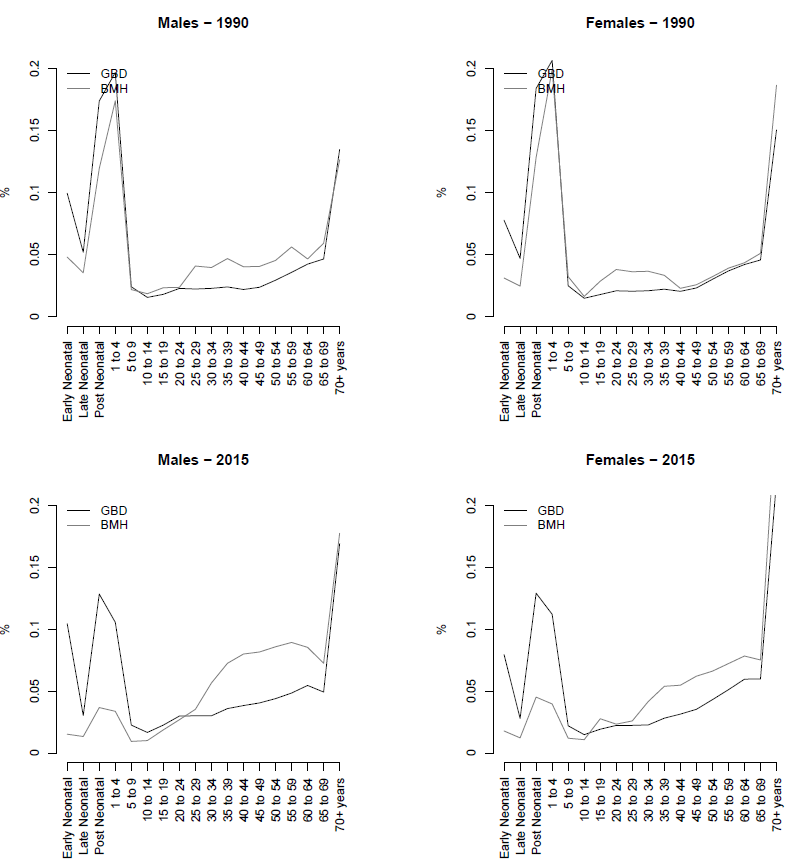


*Figure S3: Age patterns of deaths in the death notification data (BMH) for Antananarivo and in the GBD 2016 for Madagascar in 1990 and 2015*


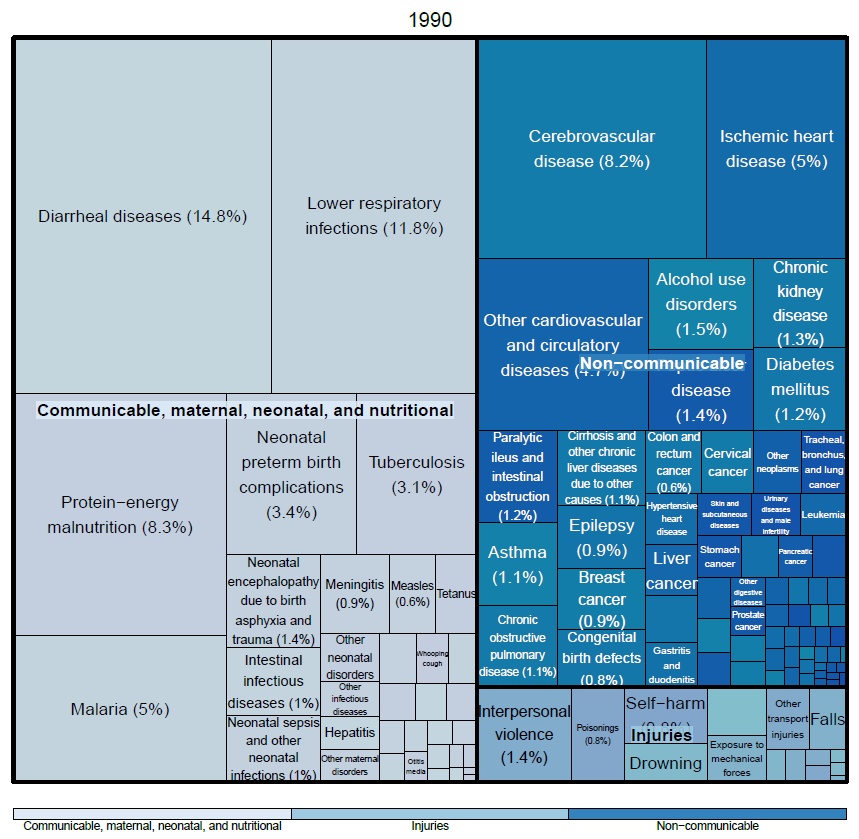


*Figure S4: Unstandardized cause-specific mortality fractions in 1990, according to the death notification data (BMH) for Antananarivo (for both sexes and all ages)*


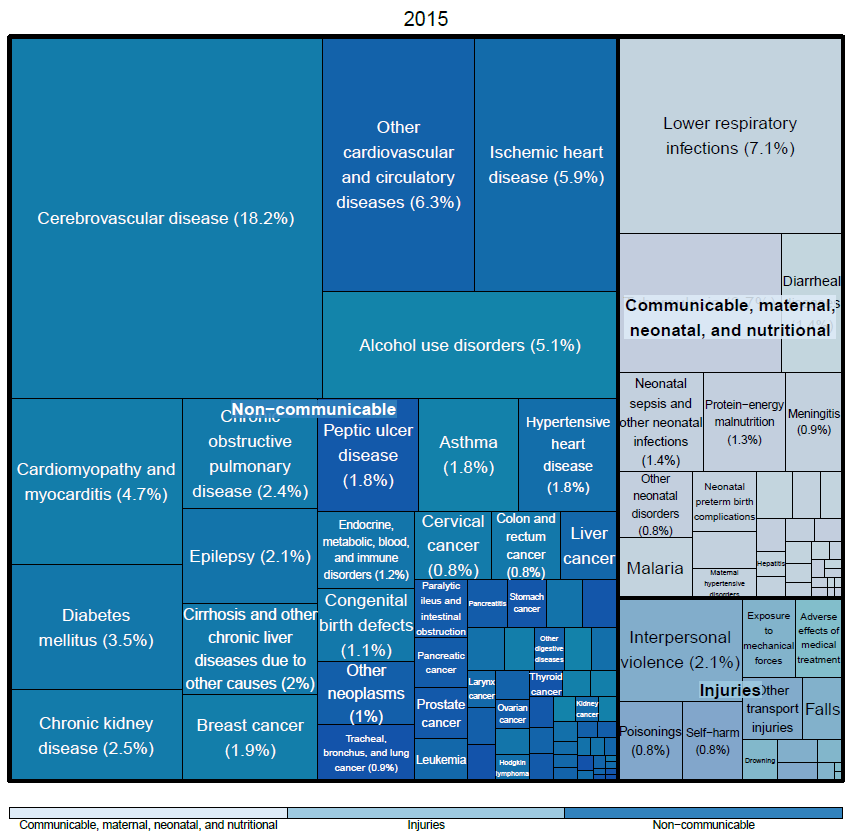


*Figure S5: Unstandardized cause-specific mortality fractions in 2015, according to the death notification data (BMH) for Antananarivo (for both sexes and all ages)*

# Computation of the composite index

In the main text, we report on each component of the index separately. In this section, we detail how we obtained a summary value from these different components. Phillips and colleagues (2014) argue that a simple combination (such as an arithmetic mean) of the different dimensions would be inappropriate because the overall quality is a function of both the level of each dimension and the cause of death composition of the population whose deaths are registered. They developed a simulation procedure to combine indicators into a single index and provide a table to transform and adjust the values of each indicator. For example, the level of garbage-coding observed in our database (27.6%, corresponding to the sum of the percentage of deaths assigned to Level 1 Garbage Codes and half the percentage of deaths assigned to Level 2 Garbage Codes) results in cause-specific mortality fractions that are 60% accurate in their simulations. Using their approach, we obtained a summary index as follows:

- *Quality of cause of death reporting*: 100-(23.8+7.6/2) = 72.4% of all deaths coded to non-garbage codes => Value of Indicator : 60%
- *Quality of age and sex reporting*: 99% => Value of Indicator: 99%
- *Medically Impossible Diagnoses:* 99% => Value of Indicator: 99%
- *Completeness of death reporting*: 92.5% => Value of Indicator: 90%
- *Level of cause-specific detail* => Value of Indicator: 100%

We refer the readers to Phillips and colleagues (2014)’s appendix for further details. The combined VS performance index for Antananarivo was obtained as 0.6 x 0.99 x 0.99 x 0.9 x 1 = 0.53. The sixth dimension (availability of timely vital statistics data) was not considered here, as the data are available upon request.

# Death report forms used by the Municipal Hygiene Office

In Madagascar, health facilities use various forms of death certificates, and the use of the World Health Organization (WHO) International Form of Medical Certificate of Cause of Death is not systematic.

The death report form used by the Municipal Hygiene Office (BMH) is reproduced below (in French).


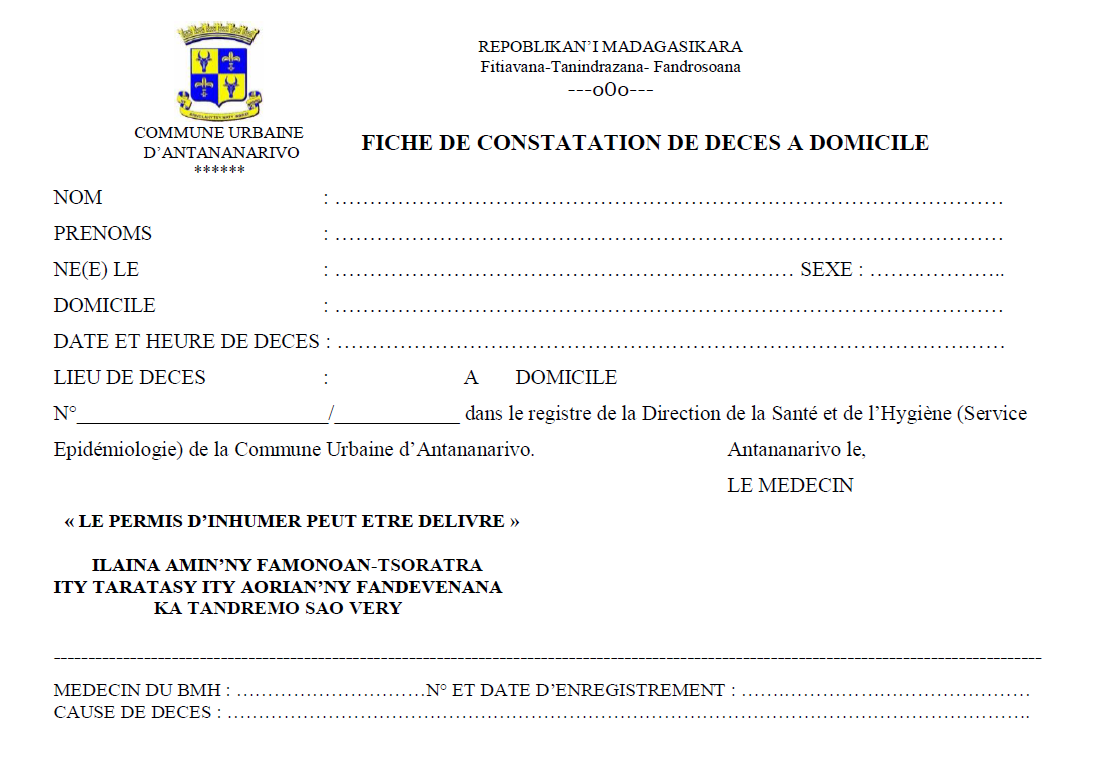


The information sheet filled by physicians in charge of certifying deaths is reproduced below.


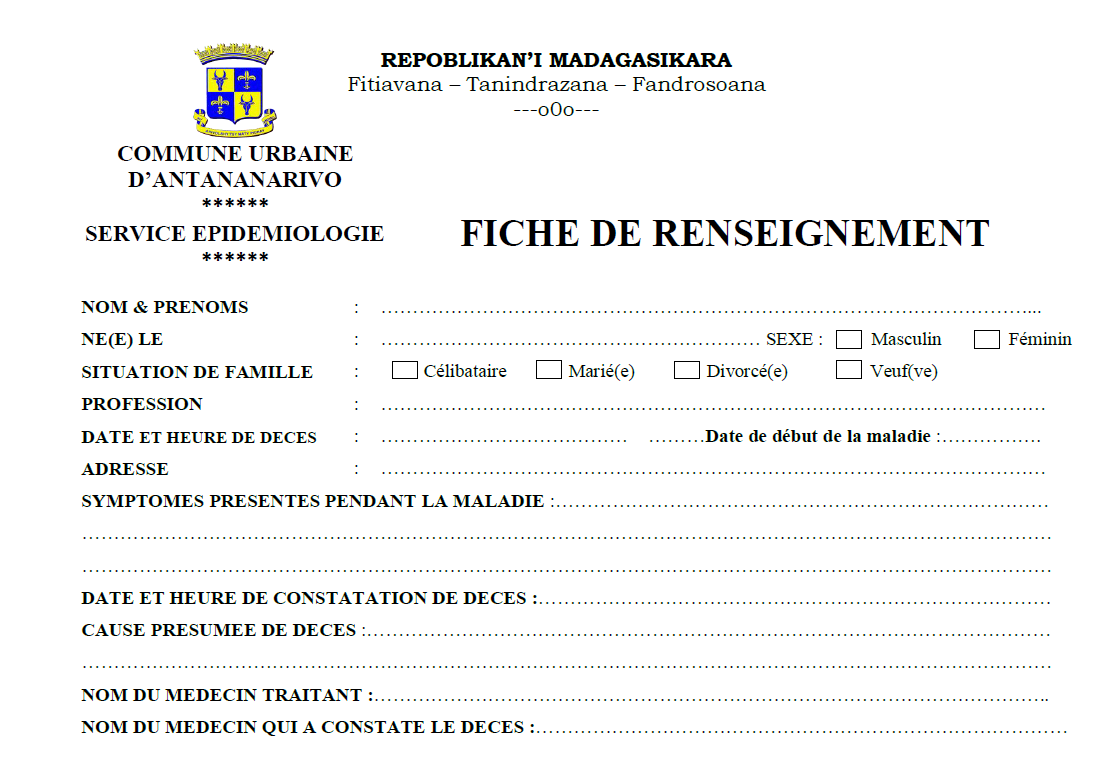


This form has been revised in 2015 in the context of influenza pandemic preparedness. It was developed by team of the virology unit at the Institut Pasteur de Madagascar (Drs Jean-Michel HERAUD and Joelinotahiana RABARISON: Project “Surveillance of mortality associated to acute respiratory infections in Antananarivo”, funded by Us Centers for Diseases Control and Prevention Cooperative Agreement # U51/IP000812). The new form is reproduced below.

**REPOBLIKAN’I MADAGASIKARA**

**Fitiavana – Tanindrazana – Fandrosoana**

**COMMUNE URBAINE D’ANTANANARIVO**

**DIRECTION DES ACTIONS SOCIALES ET DE LA SANTE INSTITUT PASTEUR DE MADAGASCAR**

**FICHE DE RENSEIGNEMENTS N° |__|__|__|__|__| / |__|__|**

Noms et Prénoms : __________________________________________________________________

Né(e) : |__|__| / |__|__| / |__|__|__|__| Age : |__|__| jour mois année Estimation Sexe : M F Situation matrimoniale : _____________ Profession : ______________________

Date et Heure de décès : |__|__| / |__|__| / |__|__|__|__| Vers |__|__| : |__|__|

Date de début de la maladie : |__|__| / |__|__| / |__|__|__|__| Date non précise : ____________

Adresse : Lot _______________ Fokontany_________________

Arrondissement |__ | District :____________________ Région :___________________

Lieu de décès : à domicile à l’hôpital  autre

Précisez si autre que son adresse à domicile : ___________________________________________

Signes d’infections respiratoires aiguës présentées pendant la maladie : Oui Non Ne sait pas

- Fièvre |__| Toux |__| Dyspnée |__|
- Autres : _________________________________________________________________________

Tests réalisés avec résultats : Oui Non Ne sait pas Nom du labo : ________________________

Date : |__|__| / |__|__| / |__|__|__|__| Date inconnue

- Germes retrouvés : Virus respiratoire syncitial |__| Grippe |__| Rhinovirus |__|

Streptocoque |__| Staphylocoque |__| Haemophilus |__| Autres : ____________________________________________________________

- Tuberculose : |__|

Autres signes présentés pendant la maladie : ______________________________________________

Comorbidités : HTA |__| Diabète |__| Cholestérolémie |__| Tabac |__| Alcool |__|

Sédentarité |__| Stress |__| Obésité |__| Asthme |__| VIH |__| Tuberculose |__| ____________________________

Autres : ______________________________________________________________

Antécédents familiaux : ______________________________________________________________

Date et heure de constatation de décès : |__|__| / |__|__| / |__|__|__|__| |__|__| : |__|__| Cause présumée de décès : ____________________________________________________________

Nom du Médecin traitant : ____________________________________________________________

Nom du Médecin qui a constaté le décès : ________________________________________________

Date et lieu de l’enterrement : |__|__| / |__|__| / |__|__|__|__| ___________________________

Nom, âge et lien de parenté du déclarant : ________________________________________________

Autres observations : ________________________________________________________________

Antananarivo le, __________________

Signature du Déclarant Le Médecin du BMH
